# Genome-Wide Identification of DNA Methyltransferases (Dnmts) in Fish and Its Potential Roles During Sex Change in Blackhead Seabream

**DOI:** 10.3390/biom15060896

**Published:** 2025-06-18

**Authors:** Sixin Guo, Binwei Duan, Jianchao Chen, Mingyang Cui, Canbei You, Hanyin Wei, Xiazi Huang, Li Deng, Kai Zhang

**Affiliations:** College of Life Sciences and Oceanography, Shenzhen University, Shenzhen 518057, China

**Keywords:** fish, DNA methyltransferases, molecular evolution, sex change

## Abstract

DNA methylation, also known as 5-methylcytosine, is an epigenetic modification that has crucial functions in multiple important biological processes in fish, such as gonadal development. The cellular DNA methylation level is tightly regulated by DNA methyltransferases (Dnmt). However, detailed investigations of this family in fish are very scarce. In this study, our results confirmed that teleost genomes contain 4 to 16 *Dnmt* genes, with diversity likely resulting from a combination of whole-genome duplication (WGD), tandem duplication, and gene loss. Differences were observed in tissue distribution, transcription abundance, and protein structure of *Dnmt* duplicates, supporting their subfunctionalization or neofunctionalization after duplication. Interestingly, we found that fish *Dnmt3b* duplicates likely have acquired the functions of mammalian *Dnmt3l*, which may compensate for the absence of fish *Dnmt3l*. Furthermore, transcriptome analysis and qPCR results indicated that DNA methyltransferase genes (*Dnmt1*, *Dnmt3aa*, *Dnmt3ab*, *Dnmt3ba*, and *Dnmt3bb.1*) possibly play important roles in the natural sex change of protandrous hermaphrodite blackhead seabream (*Acanthopagrus schlegelii*) and inferred that global remodeling of gonadal DNA methylation, regulated by DNA methyltransferase genes, was closely associated with sex change in sequentially hermaphroditic fishes. Overall, our results may help provide a better understanding of the evolution and function of DNA methyltransferases in fish.

## 1. Introduction

DNA methylation, a crucial epigenetic regulatory mechanism, plays a key role in numerous biological processes, including gene expression regulation, gene imprinting, and preservation of chromosomal integrity [1,2,3]. This process is catalyzed by a group of enzymes called DNA methyltransferases (Dnmts), which covalently add methyl groups to specific DNA sequences [4]. *Dnmt* genes were first identified as part of the restriction/modification (RM) system in 1962 [5] and have since been extensively characterized in mammals. In mammals, five *Dnmt* genes have been identified: *Dnmt1*, *Dnmt2*, *Dnmt3a*, *Dnmt3b*, and *Dnmt3l* [6]. These Dnmts function in two distinct methylation processes: maintenance (Dnmt1) and de novo (Dnmt3 subfamily, including Dnmt3a, Dnmt3b, and Dnmt3l). Dnmt1, with its higher catalytic activity on hemi-methylated substrates, primarily functions as a maintenance methyltransferase essential for genome-wide methylation maintenance [6]. De novo DNA methylation is primarily performed by Dnmt3a and Dnmt3b. Although Dnmt3l lacks catalytic activity, it plays a crucial role in enhancing the methylation functions of Dnmt3a and Dnmt3b [7,8]. *Dnmt2*, an RNA methyltransferase, predominantly targets tRNA [9].

Whole genome duplication (WGD) events result in the multiplication of chromosomes within a species [10,11,12], leading to the formation of homologous gene copies and the retention or deletion of certain genes [13,14]. This process enhances genetic diversity and complexity in vertebrates, including mammals and teleosts, thereby driving phenotypic evolution [13]. The genomes of ancestral teleosts experienced two ancient WGD events (2R) shared among all vertebrates, as well as a teleost-specific WGD, referred to as the third round of genome duplication (3R) [14,15,16]. Moreover, certain teleost species, including salmonids and some cyprinids, underwent a fourth round of genome duplication (4R) [17,18,19,20]. Consequently, teleosts possess more *Dnmt* genes than other vertebrates, with eight different *Dnmt* genes identified in zebrafish (*Dnmt1*, *Dnmt2*, *Dnmt3aa*, *Dnmt3ab*, *Dnmt3ba*, *Dnmt3bb.1*, *Dnmt3bb.2*, and *Dnmt3bb.3*) [21]. However, comprehensive investigations of *Dnmt* genes in fish are limited.

Previous studies have suggested that *Dnmts* may be closely related to gonadal development in fish [22]. In Nile tilapia (*Oreochromis niloticus*) and bluehead wrasse (*Thalassoma bifasciatum*), the expression levels of *Dnmt3aa* and *Dnmt3ab* are notably elevated in the testes compared to the ovaries and significantly increase during the sex change from female to male triggered by fadrozole [23] or social cues [24,25]. In ricefield eel (*Monopterus albus*), *Dnmt3aa* and *Dnmt3ab* exhibit high expression levels in testicular spermatocytes, with *Dnmt3aa* expression significantly increasing during the transition from female to male sex reversal [26,27]. Although the expression patterns of *Dnmts* have been reported in the gonadal development of a few gonochoristic fishes, the functional mechanisms of *Dnmts* remain largely unknown. The identification of *Dnmt* genes and investigation of their transcription profiles in a broader range of fish species would be valuable for understanding the roles of *Dnmts* in gonadal development.

Blackhead seabream (*A. schlegelii*) is one of the most popular and economically significant commercial marine fish species in China and other East Asian countries. To enhance blackhead seabream production, effective management and control of the sex ratio are critical for ensuring a sustainable supply in aquaculture systems [28]. Interestingly, the blackhead seabream is a protandrous hermaphrodite with an impressive life cycle involving natural sex change from male to female, making it an excellent model for studying the molecular mechanisms of fish sex development [29]. Nevertheless, information on DNA methyltransferases in blackhead seabream and other protandrous hermaphrodite fish is currently unavailable.

In this study, we identified genes encoding DNA methyltransferases using high-quality genome data from representative fishes, including amphibious mudskippers, deep-sea snailfish (*Careproctus pellucidus*), tetraploid *Sinocyclocheilus* fishes, salmonids, lobe-finned fish (coelacanth), hermaphrodite blackhead seabream and yellowfin seabream (*Acanthopagrus latus*), cartilaginous sharks and skates, and jawless sea lamprey (*Petromyzon marinus*) and hagfish (*Myxine glutinosa*). We further investigated the diversity, structural differences, evolutionary selection, and tissue distribution of the *Dnmt* family. Additionally, we explored the potential roles of this gene family in the natural sex transition process of blackhead seabream using transcriptome sequencing and qPCR analysis. Our results provide valuable insights into the evolution and function of the *Dnmt* family in fish, particularly in the context of sex determination and development of hermaphroditic species.

## 2. Materials and Methods

### 2.1. Sequence Collection and Characterization of Dnmt Genes

The reference sequences of *Dnmt1*, *Dnmt3ab*, *Dnmt3ba*, *Dnmt3aa*, *Dnmt3bb.1*, *Dnmt3bb.2*, and *Dnmt3bb.3* in zebrafish were downloaded from the National Center for Biotechnology Information (NCBI) to construct a local database. Whole genomes of 31 fish species were downloaded from the NCBI database. Nucleotide sequences of *Dnmt1*, *Dnmt3ab*, *Dnmt3ba*, *Dnmt3aa*, and *Dnmt3bb.1* were extracted from the blackhead seabream genome using BLAST (version 2.2.31+) [30] and GeneWise (version 2.2.0) [31]. The physicochemical properties of Dnmt proteins in blackhead seabream were analyzed using the ExPASy ProtParam tool (https://web.expasy.org/protparam/, accessed on 11 April 2024). Subcellular localization of Dnmt proteins was predicted using Cell-PLoc 2.0 (http://www.csbio.sjtu.edu.cn/bioinf/euk-multi-2/, accessed on 26 April 2024).

### 2.2. Phylogenetic Analysis, Motif Identification, and Domain Prediction

We selected 31 representative fishes with high-quality genome assembly for comparative genomic analysis. These fish species include lineages that have experienced either the fourth WGD, the third WGD, or two WGD events. *Dnmt* gene sequences from 31 bony fishes and four mammalian species (*Homo sapiens*, *Mus musculus*, *Sus scrofa*, and *Canis lupus familiaris*) from the NCBI database were used to construct phylogenetic trees and to compare the number of *Dnmt* genes across species. Phylogenetic analysis was performed using PhyloSuite software (version 1.2.3). Multiple sequence alignment was conducted using MAFFT (version 7.526) [32], and the most appropriate model was selected using ModelFinder. A phylogenetic tree was constructed using the maximum likelihood method of the IQ-TREE (version 1.6.12) tool [33], with 1000 standard replicates in the MFP model for bootstrap analysis. Gene structure and domain analyses were performed using the ChiPlot (https://www.chiplot.online/, accessed on 4 June 2024). Conserved motifs of *Dnmt* genes were identified using MEME (version 5.5.7) [34]. Protein domains were predicted using the Batch CD-Search tool of NCBI (https://www.ncbi.nlm.nih.gov/Structure/bwrpsb/bwrpsb.cgi, accessed on 9 May 2024).

### 2.3. Synteny Analysis

To evaluate the conservation of *Dnmt* genes, the genes upstream and downstream of each *Dnmt* paralog were examined. The associated region information was obtained from the GenBank database. The chromosomal locations of *Dnmt* genes in 11 representative fishes were extracted using BLAST (version 2.2.31+) [35] and GeneWise (version 2.2.0) [31]. The zebrafish genome served as the reference standard for defining the upstream and downstream regions of *Dnmt* genes.

### 2.4. Evolutionary Homology and Protein Structure of Dnmt

A phylogenetic tree of the *Dnmt* sequences from *A. schlegelii* and *A. latus* was constructed using IQ-TREE (version 1.6.12) [33]. Protein domains were predicted using InterPro [36], and Pfam predictions were used for subsequent statistical analysis. The chromosomal positions of the CDS sequences were computed using Python 3.8.17. Collinearity analysis of the two species was performed using TBTools [37]. The secondary structure of *A. schlegelii* Dnmt protein was predicted using PSIPRED (http://bioinf.cs.ucl.ac.uk/psipred/SMART, accessed on 16 May 2024) and NovoPro (https://www.novopro.cn/tools/secondary-structure-prediction.html, accessed on 21 May 2024). The tertiary structure was predicted using AlphaFold3 [38], and structural models were generated using PyMOL [39].

### 2.5. Experimental Fish

Blackhead seabream individuals were obtained from Guangdong Marine Fisheries Experimental Centre, which is located at Daya Bay in Huizhou city, Guangdong province, China. These individuals were maintained in a circulating water system at 25–28 °C with a natural photoperiod. Gonadal tissues were collected during three distinct reproductive phases: (1) the functional male phase (characterized by testis-dominant morphology, January), (2) the intersexual transitional phase (featuring testis regression with concomitant ovarian development, May–June), and (3) the functional female phase (exhibiting ovary-dominant morphology, December). For each phase, gonads from three biologically independent individuals were collected as biological replicates.

### 2.6. RNA Extraction, Quantitative Reverse-Transcription PCR (qRT-PCR) Analysis, and Transcriptomic Analysis

Total RNA was isolated from the liver, gill, spleen, intestine, stomach, and gonad (testis, ovotestis, and ovary) tissues of blackhead seabream using a SteadyPure Quick RNA Extraction Kit (AG21017). Complimentary DNA was synthesized using the PrimeScript™ RT reagent kit (Takara, Code No. RR047A) according to the manufacturer’s instructions. qRT-PCR was performed using a SYBR Green Premix *Pro Taq* HS qPCR Kit (AG11701). *β-actin* was used as an internal control. The amplification parameters were as follows: 95 °C for 30 s, followed by 40 cycles of 95 °C for 5 s, and 60 °C for 30 s. All samples were measured in triplicates. Normalization and fold change were calculated using the 2^−△△Ct^ method [40]. One-way ANOVA in GraphPad Prism 8.0 software [41] was used to analyze significant differences. The primers for all genes used in qRT-PCR are listed in Table 1. Related transcriptomic data of the tissues (testis, ovotestis, and ovary) from blackhead seabream were generated by our lab. TPM (Transcripts Per Kilobase per Million) values were used to quantify gene transcription levels.

## 3. Results

### 3.1. Genome-Wide Identification of the Dnmt Gene Family

**Figure 1:**
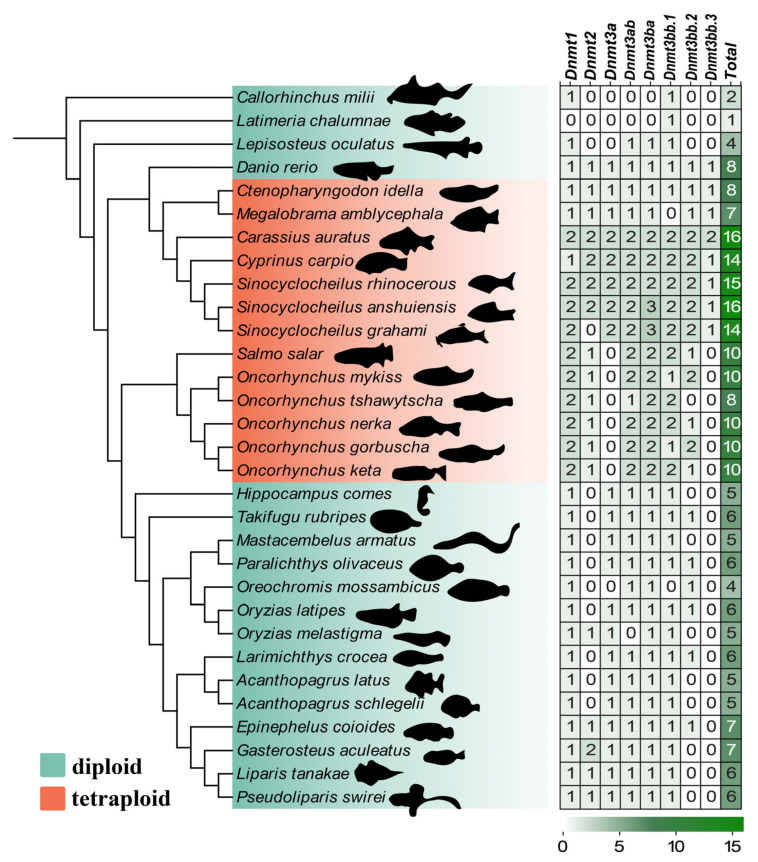
The number of *Dnmt* family members in 31 vertebrate species genomes was compared between diploid species (green in the phylogenomic tree) and tetraploid species (red in the phylogenomic tree). The higher the number of *Dnmt* genes, the darker the histogram color.

In the present study, 242 *Dnmt* genes were identified in 31 species. We confirmed that the teleosts possessed 4 to 16 *Dnmt* copies (see more details in Figure 1). In diploid teleosts, their genomes have 4–8 *Dnmt* genes. Five candidate *Dnmt* genes were identified in the protandrous hermaphrodite *A. schlegelii* genome and designated as *Dnmt1*, *Dnmt3bb.1*, *Dnmt3ab*, *Dnmt3ba*, and *Dnmt3aa*. These genes were distributed across four chromosomes (chr 23, 7, 16, and 22), with *Dnmt3bb.1* and *Dnmt3ba* sharing the same chromosomes. For the typical tetraploid teleost fishes, there are 7 to 16 *Dnmt* copies, such as river trout (*Salmo trutta*), rainbow trout (*Oncorhynchus mykiss*), and common carp (*Cyprinus carpio*), which underwent the fourth genome duplication.

Dnmt proteins ranged from 691 (Dnmt3aa) to 1508 (Dnmt1) amino acids in length. Their predicted molecular weights varied from 78.163 kDa (Dnmt3aa) to 170.325 kDa (Dnmt1), and all were classified as neutral (aliphatic index = 50–70) or hydrophobic (aliphatic index > 70). The predicted pI values ranged from 5.81 (Dnmt1) to 7.49 (Dnmt3bb.1). All five Dnmt proteins had an instability index of >40, indicating stability. Subcellular localization predictions suggested that all Dnmt members were primarily located in the nucleus and that *Dnmt3ab* and *Dnmt3aa* were also present in the cytoplasm (Table 2).

### 3.2. Phylogenetic Analysis of the Dnmt Family

**Figure 2:**
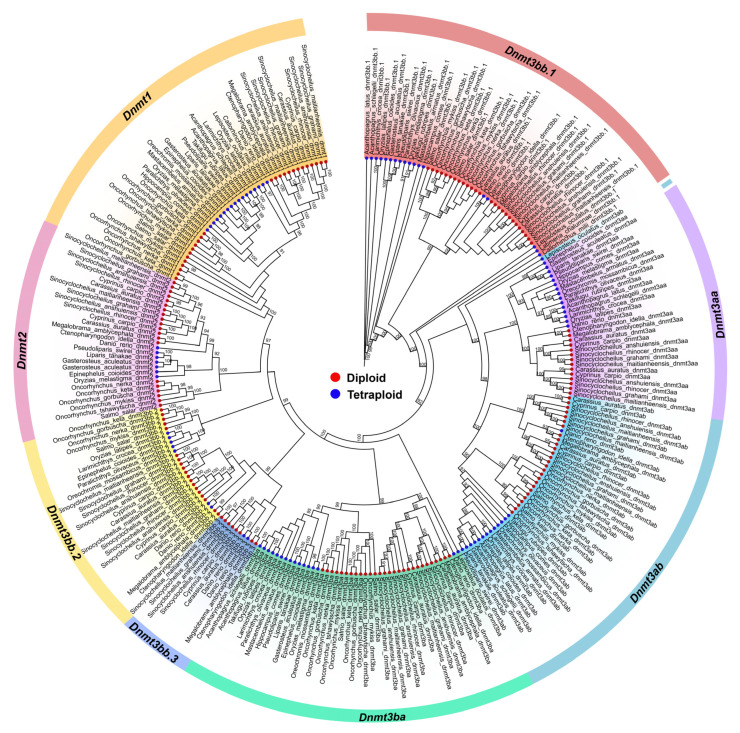
Phylogenetic tree of the *Dnmt* gene family in 31 fish species. Red and blue dots indicate diploid and tetraploid species, respectively. Orange, pink, yellow, blue, green, light blue, purple and red colors represent subfamilies *Dnmt1*, *Dnmt2*, *Dnmt3bb.2*, *Dnmt3bb.3*, *Dnmt3ba*, *Dnmt3ab*, *Dnmt3aa*, and *Dnmt3bb.1*, respectively.

A phylogenetic tree was constructed using Dnmt protein sequences from various fish species (Figure 2). *Dnmt* genes mainly clustered into eight groups (*Dnmt1*, *Dnmt2*, *Dnmt3bb.1*, *Dnmt3bb.2*, *Dnmt3bb.3*, *Dnmt3ab*, *Dnmt3ba*, *and Dnmt3aa*). *Dnmt3bb.1*, identified as a candidate gene shared among species that underwent two to three rounds of whole genome duplication (WGD) events early in vertebrate evolution (*Callorhinchus milii*, *Latimeria chalumnae*, and *Lepisosteus oculatus*) [42], formed the root of the phylogenetic tree (Figure 2). As shown in Figure 1, several species exhibited gene loss during evolution. Interestingly, *Dnmt3bb.3* formed a distinct clade within Cyprinidae. Within each *Dnmt* subfamily, the Dnmt3a and Dnmt3b groups were generally clustered closely, suggesting similar biological functions consistent with previous studies [4,43]. In contrast, Dnmt1 was far more distantly related, indicating a functional divergence from Dnmt3a/3b.

### 3.3. Synteny Analysis

**Figure 3:**
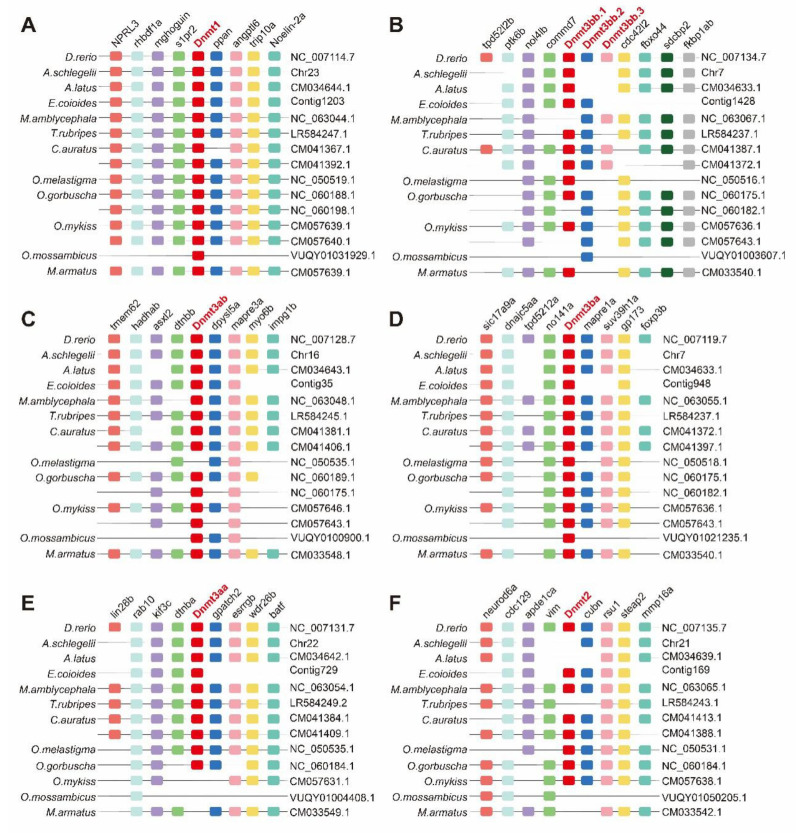
Syntenic analysis of chromosomal loci containing *Dnmt* genes in various fish species. Colored rectangles represent distinct loci, with some gene loci adjacent to *Dnmt* showing conserved synteny across the examined species. Panels (**A**–**F**) correspond to *dnmt1*, *dnmt3bb*, *dnmt3ab*, *dnmt3ba*, *dnmt3aa*, and *dnmt2*, respectively. In panels (**A**,**C**–**F**), red blocks represent different *dnmt* subtypes. In panel (**B**), the red, blue, and pink rectangles represent *dnmt3bb.1*, *dnmt3bb.2*, and *dnmt3bb.3*, respectively. Species names are shown on the left, and chromosome numbers are labeled on the right. (*D. rerio*, *Danio rerio*; *A. schlegelii*, *Acanthopagrus schlegelii*; *A. latus*, *Acanthopagrus latus*; *E. coioides*, *Epinephelus coioides*; *M. amblycephala*, *Megalobrama amblycephala*; *T. rubripes*, *Takifugu rubripes*; *C. auratus*, *Carassius auratus*; *O. melastigma*, *Oryzias melastigma*; *O. gorbuscha*, *Oncorhynchus gorbuscha*; *O. mykiss*, *Oncorhynchus mykiss*; *O. mossambicus*, *Oreochromis mossambicus*; *M. amatus*, *Mastacembelus armatus*).

To evaluate the synteny of conserved genes upstream and downstream of *Dnmt* in various fish species, we examined the collinear relationship between these conserved genes and *Dnmt*. As shown in Figure 3, *Dnmt1* shared a conserved suite of flanking genes, whereas other *Dnmt* genes (*Dnmt3bb.1*, *Dnmt3bb.2*, *Dnmt3bb.3*, *Dnmt3ab*, *Dnmt3ba*, and *Dnmt3aa*) exhibited gene loss during evolution. *O. mossambicus* displayed extensive gene loss, including *Dnmt* genes and nearby loci, suggesting potential functional loss or compensation by other genes. *Dnmt3bb.1*, *Dnmt3bb.2*, and *Dnmt3bb.3* were closely repeated on the same chromosome in some species such as *D. rerio* and *C. auratus* (Figure 3B). However, the partial loss of these genes and nearby loci was observed in other fish species. Among these fishes undergoing a fourth whole genome duplication event, *C. auratus*, *O. gorbuscha*, and *O. mykiss* possessed two copies of certain *Dnmt* genes (*Dnmt1*, *Dnmt3bb.1*, *Dnmt3bb.2*, *Dnmt3bb.3*, *Dnmt3ab*, and *Dnmt3ba*) on separate chromosomes (Figure 3A–D).

### 3.4. Structural Analysis of Dnmt Proteins

**Figure 4:**
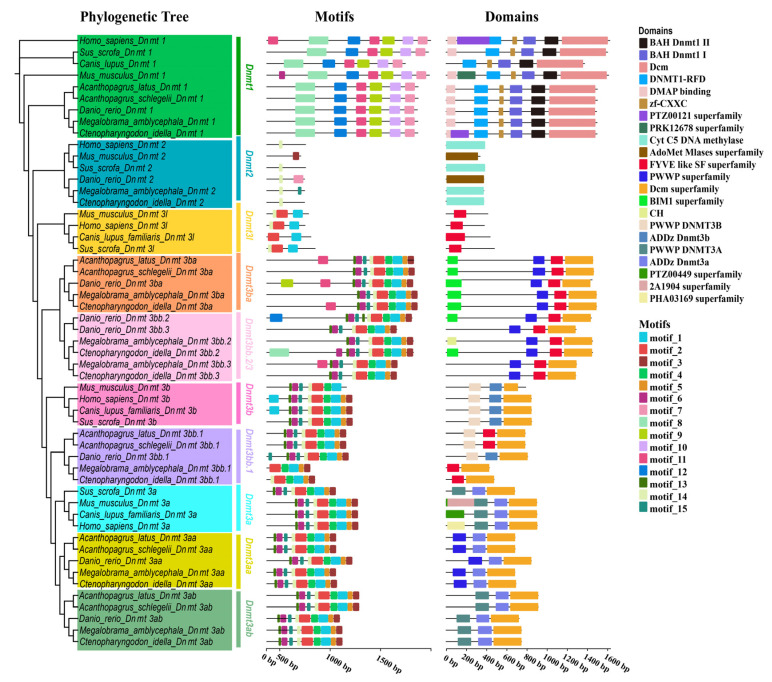
Phylogenetic relationships, gene structures, and motifs of *Dnmt* genes.

A phylogenetic tree was constructed using Dnmt proteins from four mammals (Homo sapiens, Mus musculus, Sus scrofa, and Canis lupus familiaris) and five bony fishes (Ctenopharyngodon idella, Megalobrama amblycephala, Danio rerio, Acanthopagrus schlegelii, and Acanthopagrus latus) (Figure 4). The analysis revealed that four Dnmt proteins (Dnmt1, Dnmt2, Dnmt3b, and Dnmt3a) were present in the four vertebrates, and five to eight Dnmt proteins were found in the five fish species. Shared motifs and domains showed slight differences among the species. All Dnmt1 proteins contained BAH_Dnmt1_I, BAH_Dnmt1_II, and Dcm domains, with conserved motifs 7–12. Dnmt2 proteins possessed motifs 3, 4, 7, and 14 and two domains (Cyt_C5_DNA_methylase and zf_CXCX). All Dnmt3 proteins (Dnmt3l, Dnmt3ba, Dnmt3bb.2, Dnmt3bb.3, Dnmt3a, Dnmt3aa, and Dnmt3ab) shared motifs 1–2. Except for Dnmt3l, the other Dnmt3 proteins contained motifs 1–5 and the Dcm superfamily. In summary, Dnmt proteins were classified into four types: Dnmt1, Dnmt2, Dnmt3l, and Dnmt3b/a. Within each type, the proteins exhibited highly similar structures, suggesting potentially similar biological functions.

### 3.5. 3D Structures of Dnmts in Blackhead Seabream

**Figure 5:**
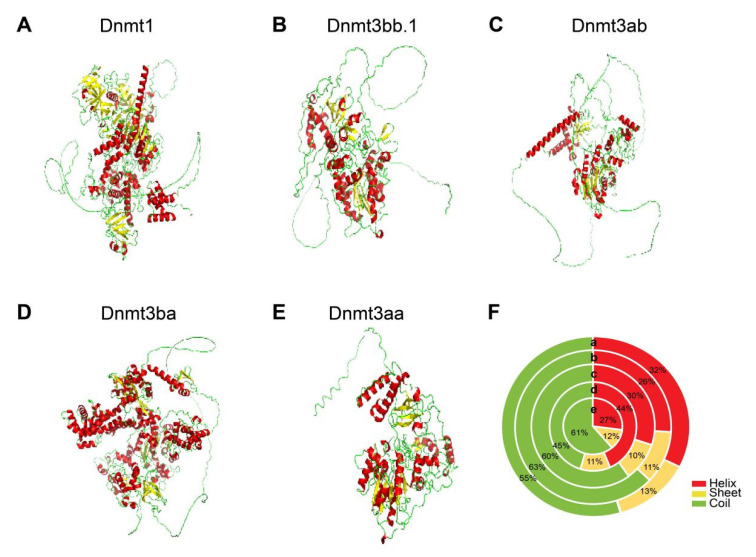
The 3D structure modeling of Dnmt proteins. The red color represents α-helix, yellow represents β-sheet, and green represents coil. The protein structures were predicted by AlphaFhold3, and the structure image was generated using the pymol software (**A**–**E**). The pie chart shows the statistical proportion of protein secondary structure (**F**) (a. *Dnmt1*, b. *Dnmt3bb.1*. c. *Dnmt3ab*, d. *Dnmt3ba*, and e. *Dnmt3aa*).

The 3D structures of the five Dnmt proteins were predicted using AlphaFold3 (Figure 5). All modeled structures contained α-helices, β-sheets, and coils. The β-sheet content ranged from 10 to 13% for all five proteins. Except for β-sheet, Dnmt1 and Dnmt3ba showed a higher α-helix content and fewer coils, whereas Dnmt3bb.1, Dnmt3ab, and Dnmt3aa showed the opposite pattern.

### 3.6. The Expression Profile of Dnmts in Different Tissues

**Figure 6:**
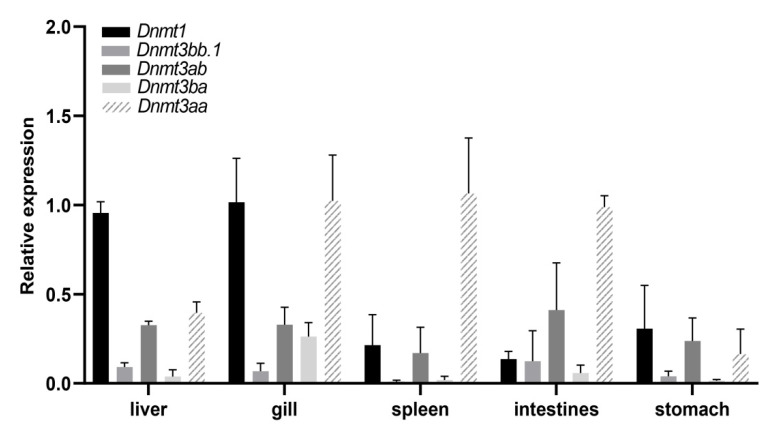
Expression profiles of *Dnmt* genes in different tissues of *A. schlegelii* were analyzed by quantitative real-time PCR (qRT-PCR). Relative expression levels of *Dnmt* genes in the liver, gill, spleen, intestines and stomach, with β-actin as an internal control.

The expression profile of *Dnmt* genes of blackhead seabream in five tissues (liver, gill, spleen, intestines, and stomach) was investigated by qRT-PCR analysis (Figure 6). Our results showed that the *Dnmts* were expressed in all the investigated tissues at varying levels. The *Dnmt1* gene was highly expressed in the gill and liver but lowly expressed in the spleen, intestines, and stomach. The expression level of *Dnmt3ba* in the gill was higher than that in other tissues. *Dnmt3ab* was expressed in all five tissues at similar levels. *Dnmt3bb.1* showed low expression across all five tissues. In addition, *Dnmt3aa* was highly expressed in the gills, spleen, and intestines but lowly expressed in the liver and stomach.

### 3.7. The Expression Profiles of Dnmts During the Natural Sex Change of Blackhead Seabream

**Figure 7:**
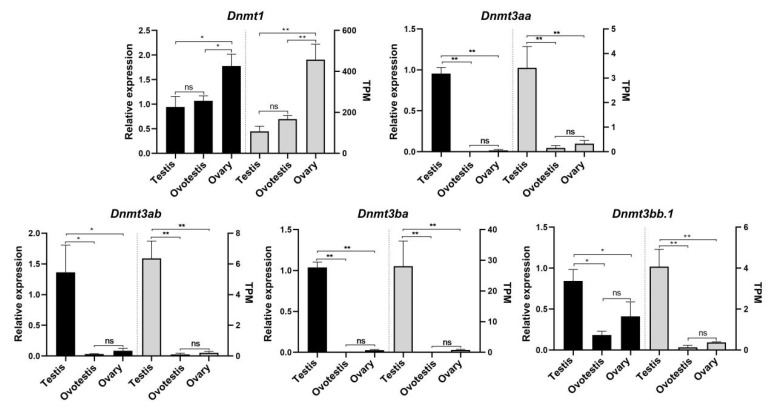
The expression patterns in testis, ovotestis, and ovary of *A. schlegelii* based on qRT-PCR (left) and RNA-seq (right). Column charts and significance analysis were constructed by Prism 8.0. The * indicates significant differences by one-way ANOVA (ns: not significant, *p* > 0.05; * *p*  <  0.05; and ** *p*  <  0.01; n = 3).

We analyzed the expression profiles of *dnmt* genes during the natural sex change of blackhead seabream based on transcriptomic data and qPCR data. Our results demonstrated that these datasets generated by the two separate methods are typically in agreement (Figure 7). Intriguingly, all the *Dnmt* genes exhibited significant sex-biased expression patterns. *Dnmt1* was significantly more highly expressed in the ovary compared to the ovotestis and testis (*p* < 0.01), whereas the other four *Dnmt* genes (*Dnmt3bb.1*, *Dnmt3ab*, *Dnmt3ba*, and *Dnmt3aa*) exhibited higher expression in the testis than in the other two developmental stages (*p* < 0.01). Notably, *Dnmt1* displayed a progressive and significant upregulation in expression during the natural sex change of blackhead seabream. Moreover, *Dnmt3ab*, *Dnmt3ba*, and *Dnmt3aa* showed almost no expression in the ovotestis and ovary.

## 4. Discussion

Gene duplication not only plays a pivotal role in fish evolution but also provides raw material for functional innovation [17]. Two dominant mechanisms are considered to generate duplicated genes, including whole genome duplication (WGD) and tandem duplication resulting from unequal crossing over [44,45]. Mammals possess five *Dnmt* genes (*Dnmt1*, *Dnmt2*, *Dnmt3l*, *Dnmt3a*, and *Dnmt3b*) [6], whereas the investigated diploid fishes have up to eight *Dnmt* isoforms (*Dnmt1*, *Dnmt2*, *Dnmt3aa*, *Dnmt3ab*, *Dnmt3ba*, *Dnmt3bb.1*, *Dnmt3bb.2*, and *Dnmt3bb.3*), and tetraploid teleost fish like salmonids and certain cyprinids have even more copies of *Dnmt* genes resulting from fish-specific WGD events. Like mammals, most fish have only one *Dnmt1* and one *Dnmt2*, while *Dnmt3l* is lost in the fish genome, suggesting that fish *Dnmt1* and *Dnmt2* are highly conserved during evolution. However, *Dnmt3* underwent a lineage-specific evolution. Moreover, the ratio of the *Dnmt* gene number between diploids and tetraploids is not always 1:2, possibly due to selective gene loss. We analyzed gene duplication types among fish *Dnmt* genes and found that *Dnmt3bb.1*, *Dnmt3bb.2*, and *Dnmt3bb.3* were tandemly and intrachromosomally duplicated, suggesting their origin of independent and continuous duplication. Our findings demonstrated the significance of tandem gene duplication as well as that of WGD in the course of *Dnmt* evolution, and copy number variations of the *Dnmt* gene likely resulted from WGD, tandem duplication and gene loss.

Duplicate genes typically follow two evolutionary trajectories: subfunctionalization (partitioning ancestral functions) or neofunctionalization (acquiring novel functions) [46]. For *Dnmts*, the presence of putative duplicates in most fish species suggests that some subfunctionalization or neofunctionalization occurred within the *Dnmt* family. *Dnmt1* is localized to replicating DNA and heterochromatin through interactions with PCNA and UHRF1, as well as by direct binding to heterochromatic histone modifications H3K9me3 and H4K20me3 [41]. Dnmt3 enzymes bind to heterochromatin via protein multimerization and are targeted to chromatin by their ADD, PWWP, and UDR domains, which bind to unmodified H3K4, H3K36me2/3, and H2AK119ub1, respectively [41]. In the present study, differences in the number and type of domains were identified among fish *Dnmt* isotypes, potentially contributing to functional diversity and complexity. Furthermore, all the *Dnmt3* duplications retained the ADD, PWWP, and Cyt_C5_DNA_methylase domains, suggesting similar functional capabilities. In mammals, de novo DNA methylation is mediated by *Dnmt3a* and *Dnmt3b* with the assistance of the stimulatory factor *Dnmt3l* [41]. Compared to mammals, the DNA methylation mechanism in fish is likely more complex because of the multiple *Dnmt3* duplicates and the absence of *Dnmt3l* in their genome [45]. In mammals, the de novo methyltransferases Dnmt3a and Dnmt3b share similar structural compositions, both containing a PWWP domain, an ADD domain, and a C-terminal catalytic domain. In contrast, Dnmt3l lacks both the PWWP domain and the catalytic domain, while Dnmt1 possesses a C-terminal domain, two BAH domains, and a CXXC domain. Our findings demonstrate that mammalian Dnmt3 genes uniformly retain both PWWP and ADD domains, whereas teleost Dnmt3 and its homologs exhibit either one or both domains. This suggests lineage-specific diversification of Dnmt3 subfamily members during fish evolution, implying potential functional innovation or subfunctionalization in teleost DNA methylation catalysis. Interestingly, fish *Dnmt3b* duplicates contain the FYVE-like SF superfamily domain, which is the only domain present in mammalian *Dnmt3l*. We hypothesize that fish may have evolved a DNA methylation mechanism distinct from that of mammals, with *Dnmt3* duplicates potentially replacing the function of mammalian *Dnmt3l*.

To better understand the functions of *Dnmts* in fish, we examined their distribution across various tissues in blackhead seabream. *Dnmt1*, *Dnmt3aa*, and *Dnmt3bb* genes were highly expressed in the liver, gill, spleen, intestine, and stomach, indicating their important roles in these tissues. Additionally, *Dnmt* copies exhibited different expression patterns among the examined tissues, which was consistent with previous studies in zebrafish [21] and tilapia [47]. These findings support the view that *Dnmt* copies have undergone subfunctionalization or neofunctionalization following duplication [48].

DNA methylation likely underwent remodeling at intermediate phases of sex change in the fish gonad, a process mediated by DNA methyltransferase [24]. In the present study, DNA methyltransferase genes exhibited a turnover in sex-specific expression during natural sex change in blackhead seabream gonads. Our results revealed that the maintenance methyltransferase gene *Dnmt1* exhibited female-biased expression that significantly increased during the sex change from male to female, whereas de novo DNA methyltransferase genes (*Dnmt3aa*, *Dnmt3ab*, *Dnmt3ba*, and *Dnmt3bb.1*) have highest expression at the male stage in protandrous blackhead seabream. It was previously demonstrated that the sex transition from male to female in blackhead seabream occurred alongside a significant decline in *cyp19a1a* (a crucial female-related gene) methylation in the ovary [29,48]. Therefore, we speculated that the DNA methylation landscape in gonads was reconfigured as the expression of female-specific *Dnmt* superseded male-specific expression in blackhead seabream. A similarly distinct sex-specific expression pattern of *Dnmt* genes has recently been reported in gonadal transcriptomes of other teleosts undergoing sex change, whether through natural processes [24,27] or experimental induction [49,50]. Furthermore, earlier findings showed that changes in DNA methylation were closely related to sex change in sequentially hermaphroditic fishes, including protogynous orange-spotted grouper [51], rice-field eel [26,52], and protandrous barramundi [53]. Taken together, global reprogramming of gonadal DNA methylation, regulated by DNA methyltransferase genes, might be a convergent feature during sex change in hermaphroditic fishes.

## 5. Conclusions

This study offers a comprehensive analysis of the *Dnmt* gene family in fish and explores their roles in natural sex change in protandrous hermaphrodite blackhead seabream. We confirmed the presence of at least 21 *Dnmt* genes in fish, and a combination of WGD, tandem duplication, and gene loss was likely responsible for the diversity of *Dnmt* genes in fish. Differences were also observed in tissue distribution. Furthermore, protein sequence alignments and structural analysis of fish *Dnmt* duplicates supported their subfunctionalization or neofunctionalization. Our data revealed that *Dnmt* genes (*Dnmt1*, *Dnmt3aa*, *Dnmt3ab*, *Dnmt3ba*, and *Dnmt3bb.1*) possibly play important roles in the natural sex change process in blackhead seabream. We inferred that DNA methyltransferase genes likely regulated the remodeling of gonadal DNA methylation during sex change in sex-changing fishes. In summary, our findings will benefit further functional investigations of these fish genes.

## Figures and Tables

**Table 1:** The primer sequence used for qPCR.

Primer	Forward Primer (5′-3′)	Reverse Primer (5′-3′)
*Dnmt1*	TCCCACAGCACAAGATTACA	AGGAACACCACCATCCAAGC
*Dnmt3bb.1*	CCGTTCTTCTGGCTGTTCG	TTCTGGGAGGCTGTGATGG
*Dnmt3ab*	ATGTCAGCCTTGAGCACCCG	TCGTCCTCCGCAGCAGATAG
*Dnmt3ba*	CCCTGGCATGAACAGACCC	CCATCTTGCCCTGCCGTAT
*Dnmt3aa*	CTCCTGCGGAAGCCTCAAT	CTGGTAGCCGTCATCGTCAT
*β-actin*	ACAGGGAGAAGATGACCCAGAT	CACCGGAGTCCATGACGATA

**Table 2:** The Dnmt physical and chemical properties in blackhead seabream.

Protein	Chr	Number of Amino Acids	Molecular Weight	Theoretical pI	Instability Index	Aliphatic Index	GRAVY	Predicted Location
*Dnmt1*	23	1508	170,325.94	5.81	47.95	65.79	−0.701	Nucleus
*Dnmt3bb.1*	7	792	89,338.44	7.49	46.73	70.29	−0.509	Nucleus
*Dnmt3ab*	16	921	104,840.84	7.24	61.31	63.6	−0.72	Nucleus, cytoplasm
*Dnmt3ba*	7	1471	166,048.65	6.87	44.89	76.51	−0.404	Nucleus
*Dnmt3aa*	22	691	78,163.04	6.05	51.93	69.29	−0.431	Nucleus, cytoplasm

## Data Availability

The original contributions presented in this study are included in the article. Further inquiries can be directed to the corresponding authors.
